# Extra Supplement.—The Nursing Mirror

**Published:** 1893-12-02

**Authors:** 


"The Hospital" Nursing Mirror.

Being the Extra Nursing Supplement of "The Hospital" Newspaper.

[Contributions for this Supplement should be addressed to the Editor, The Hospital, 428, Strand, London, W.C., and should have the word "Nursing" plainly written in left-hand top corner of the envelope.]

### News from the Nursing World.

### CHRISTMAS FESTIVITIES.

Our friends are beginning to talk of "Christmas Entertainments," in fact at one hospital, at least, an early date is already fixed for the patients' fête. It is therefore time for us to remind our readers that due notice of festivities and descriptions intended for insertion in The Hospital must reach the office in good time. We are delighted to spare space in our columns for chronicling as many as possible of the entertainments given to patients and nurses, but naturally they are only suitable for the Christmas season, and we cannot permit them to exceed certain definite limits. It will therefore be of mutual advantage if all members of the large circle of our correspondents will aid us by sending in their contributions immediately after each event. Any approaching festivities of which preliminary announcements are desired must be officially reported to us ten days at least before they are to take place.

### OUR CHRISTMAS COMPETITIONS.

Will our readers kindly tell their friends that the garments sent in for our Christmas Competition are distributed exclusively to the adult sick poor who spend the festival in hospital wards? Each year more requests are received than can possibly be satisfied, although we have the pleasure of handing over many very delightful parcels. The needlework being expressly done for poor hospital patients, as has been set forth in each paragraph, it is quite impossible to divert it into other channels. This will sufficiently explain our inability to comply with the requests sent from all classes of institutions devoted to different, though, doubtless, to most deserving objects. We anticipate that an immense variety of garments have this year been made by our readers for the adult sick poor who have to spend their Christmas Day within hospital walls. All parcels should reach The Hospital Office, 428, Strand, in the course of next week, bearing the words "Needlework Competition" in left-hand corner of address label, and also the name of the sender.

### FAIR PAY.

"What do you consider fair pay?" is a rather common retort to the thoughtful person who ventures to broach the subject of the adequate remuneration due to women's work. When unskilled labour is in question, when, in fact, some single woman is suddenly driven to earn her own living without preparation or any special aptitude for doing so, we should certainly say she must take anything she can get. When, however, a thoroughly qualified nurse is in question, it is quite another thing, and the varying rate of value placed upon her services in different quarters is most incomprehensible. Only last week the Dunmow Union, Essex, offered £30, washing, and rations to a trained nurse; the Fulham Fever Hospital, £36; whilst the Chelsea Hospital for Women, hopes to get for £20 per annum, and indoor uniform, a staff nurse to take charge of theatre, and who must have had previous experience in gynœcological work.

### THOROUGHLY TRAINED.

The Nurses' Co-operation, 8, New Cavendish Street, certainly deserves all the success which has attended it, no pains being spared in selecting new members as suitable nurses for all classes of cases. It is often asked what qualifications are required in a candidate? Briefly, then, she must hold a certificate from an accredited training school, and must have experience in private nursing. More than 400 candidates applied for the November election, and only 109 of these had undergone the requisite amount of training, and 71 were passed. For the services of fully-qualified nurses holding good certificates and satisfactory testimonials there is an increasing demand, but it is useless for those to apply who have only had one year's training, or who have merely worked in special institutions. Valuable as special branches of nursing often prove, they must invariably be supplemented by a thorough training in general nursing to qualify a nurse for joining that excellent association, the Nurses' Co-operation.

### HER LUGGAGE.

"Has Nurse come?" "Yes, Doctor, she's come, and such a lot of luggage!" replies the flushed maid who has been struggling to fit two large trunks into the small dressing-room, the only available spot in a house already disorganised by its mistress's serious illness. The doctor looks surprised, but feeling that it is not his concern, he proceeds to see the patient. Truly the number and weight of the trunks which some private nurses take about with them is causing much needless annoyance to employers, who consider rightly that the entire outfit of uniform and personal necessities could be contained in a very moderate-sized box—such a box as would take but little room, and be easily moved—in fact just the one which the best nurses always content themselves with. It is the thoughtless, selfish, or at any rate inconsiderate woman who arrives at "a case" improperly encumbered and causes dismay instead of relief at her advent. The trunks which contain stores of private unprofessional raiment and sundry other treasures, dear to the heart of their owner, have nothing whatever to do with her immediate work, and, therefore, she has no right whatever to inconvenience with them the household to whose service she is temporarily pledged.

### WANTED, A WELL-QUALIFIED NURSE.

Under the heading, "One Nurse Only," The Hospital of April 22nd called attention to the fact that although the Coleraine Guardians had advertised for both a nurse and assistant nurse, they eventually only engaged the former, and at the instigation of a member of the Board decided to do without the second. The matter has recently been taken up again, as there was a prevailing impression that if the Guardians did not act, the Local Government Board probably would. No wonder that the Medical Officer gave it as his opinion that a permanent assistant infirmary nurse was a necessity. Amongst the cases under treatment during the last three months he records a fractured base and a case of severe nervous disease, both patients requiring constant watching. The Board eventually decided to advertise for a nurse, and to offer her £15 per annum. It would have been better if the "untrained" but "well-qualified" assistant the Guardians hope to secure had been engaged in April, as she might by this time have learnt something of the duties and responsibilities attending the care of the sick and the infirm.

### QUEEN'S NURSE AT SELKIRK.

The Queen's nurse at Selkirk is highly valued by her patients, and the doctors testify to her skill and kindly tact. A large number of serious cases have been successfully nursed in their own homes during the year, and six convalescents have been sent to the Homes at Dunoon and Largs. The friends of the invalids must be proud to read that "the pleasant bearing of the Selkirk patients and their strict attention to the rules of the house have been favourably commented on by the authorities in these homes." The funds of the association are in a satisfactory state, and certainly appear to be very wisely administered. Much helpful interest is shown by all classes in the nursing scheme, and many are the useful gifts received by Nurse Smith for the use of her poor patients.

### AN ELOQUENT APPEAL.

The Archbishop of Dublin made a most eloquent speech on November 20th, at the annual meeting at St. Lawrence's Catholic Home, and aroused much general interest in the work of this branch of Queen Victoria Jubilee Institute in Ireland. He spoke with hearty commendation of the plan of the work, and plainly told his hearers that admiration of the nurses' labours was by no means equivalent to helping them, and that liberal pecuniary assistance was wanted. Lord Meath and Lady O'Hagan were amongst those present, both members of the Central Council of the Institute. Miss St. Clair's able management was cordially acknowledged, and the excellence and extent of the work done by the four nurses. An urgent appeal was made for Catholic ladies to come and be trained for nursing the sick poor in their own homes. The Archbishop illustrated his appeal by a story of two Little Sisters of the Poor who called on a good lady who had, they heard, praised their work to all her friends. She listened to their account of their projects with evident pleasure, and "they waited for the generous response which they felt sure would come as the result of their little appeal." After a lavish outpouring of words the lady declared her inability to give the clothes, money, or food which the Little Sisters wanted; but, feeling she must do something, she recommended to their care and charity two excellent poor women who wanted a home. "This was the extent of her co-operation," concluded the Archbishop drily, amidst the laughter of his hearers.

### TRAINING AT PHILADELPHIA.

The managers of the University Hospital at Philadelphia, after much thought and deliberation, have decided to lengthen the course of training to three years, instead of two as formerly. The idea of the post graduate course is abandoned, and it is proposed instead to make the three years' term of training cover housekeeping, cooking, office and laundry work as well as the usual instruction in theoretical and practical nursing. As this will necessarily entail additional expense on the hospital, a part of the cost has been cheerfully undertaken by the lady visitors, who have always entered most heartily into every scheme for the elevation and advantage of the nursing profession.

### INDIA.

A good site having been secured for the new hospital at Patna, the foundation-stone has been laid by the Lieutenant-Governor of Bengal. Five fully-qualified medical women are working in the stations of Lucknow, Benares, and Patna, and five more are training to join them. It is most satisfactory to find that the demand for fully (not half) qualified women doctors and thoroughly (instead of insufficiently) trained nurses is becoming generally understood by those who labour for the welfare of Indian women. Dr. Grace Mackinnon reports that the dispensary at Patna is highly valued by the natives, who come there from long distances, and arrive in a somewhat cleaner and quieter condition than of old. They begin to understand and comply with the necessary regulations as to patients being treated in order, &c. Although there is still something to be desired on these points, still it is pleasant to read that "we now have less trouble with a hundred than we had formerly with twenty patients."

### INFLUENZA IN VICTORIA.

Our old enemy influenza does not confine its attack to any class or any country. We hear of a recent serious visitation at the Austin Hospital for Incurables in Victoria, where many of the patients and two-thirds of the staff were prostrated by the complaint. The Austin Hospital is most picturesquely situated on a hillside in the suburb of Heidelberg, about eight miles from Melbourne.

### SHORT ITEMS.

Ninety invalids have sailed for Netley in the "Euphrates," which left Bombay early in November. —A suggestion was made at a recent meeting of the Newport Guardians that a certificated assistant nurse should be engaged for the union infirmary.—Ardwick and Ancoats District Nursing Society has received valuable gifts of clothing from the Manchester District Hospital Guild and the S.E. Lancashire Needlework Guild.—*Cassell's Saturday Journal* of November 22nd contains a very sensible article headed, "Why Women Become Hospital Nurses."—Lord Londonderry has offered to give a cottage and a subscription of £25 per annum towards the support of a district nurse at West Rainton.—The Medical Officer has reported to the Canterbury Board of Guardians that several old people want looking after, and should have a night nurse. But the Board have contented themselves with appointing an assistant nurse to do night nursing or "anything the Board wished." This is hardly helpful to the doctor, who is well aware that only a permanently appointed and qualified night nurse can give to the sick and infirm proper attention. However, the Guardians decided to let the nursing go on in the usual manner for three months. Perhaps, in the meantime, some champion may arise with moral courage to advance the claims of the sick, the friendless and the dying, to adequate night watching.

### On the Nursing of Diseases of the Stomach.

### IV.—HÆMATEMESIS.

Hæmatemesis is vomiting of blood. But when there is blood in the stomach, it may not all be got rid of by vomiting, but some of it may pass on through the pyloric orifice into the duodenum, and so along the intestines and colour the stools. The blood in its passage through the intestines becomes much altered, so that when mixed with the fæces it does not appear red, but very dark, often perfectly black or "tarry." Indeed, the whole of the blood may pass this way and none of it be vomited. A patient may really be passing blood and yet be unaware of doing so, failing to recognise its presence. On the other hand, the dark slatey colour of the fæces when iron or bismuth is being taken must be distinguished from the colour due to blood.

It may appear at first sight as if there would be no difficulty in saying whether blood has been vomited or coughed up, whether it has come from the stomach—that is hæmatemesis—or whether it has come from the lungs—that is hæmoptysis. Yet at times it is difficult to be certain which it is, especially if the patient's account is all that there is from which to judge. For when blood is vomited from the stomach a cough may be excited at the same time, and the patient be misled into thinking he has coughed up blood. Or when blood is brought up by a cough some of it may be swallowed and give rise to vomiting, or the act of coughing may lead to retching. Thus, owing to the possible fallacies, it will be useful to consider the differences between hæmoptysis and hæmatemesis.

In the first place, the stomach being a cavity, bleeding takes place into it before vomiting occurs, and there may be symptoms of loss of blood before the hæmatemesis. Such symptoms are pallor, faintness, giddiness, dimness of vision. On the other hand, hæmoptysis takes place suddenly, and the patient is aware of it by feeling the blood in his throat or mouth, and subsequently he may become faint from loss of blood if there be much hæmoptysis.

Secondly, if any blood is left in the stomach it will go downwards through the intestines, and colour the stools as explained. If there is any blood left in the air passages the expectoration for some little time—at any rate, for some hours, it may be for some days—will be discoloured by admixture with blood.

Thirdly, blood that comes from the stomach is mixed with gastric juice, which makes it acid and dark coloured, and it also may be mixed with the particles of food in the stomach. The appearance of the matter vomited is often described as being like coffee grounds. If the bleeding into the stomach has been very rapid and in large quantities, the vomit may be bright red and fluid, but as a rule it is solid, having become clotted. On the other hand, blood that is coughed up from the lungs is not acid, it is bright red and frothy from having been mixed with air. Also it is not brought up in such large quantities at one time, from the lungs as from the stomach.

*Causes of Hæmatemesis.*—The most common cause is an ulcer of the stomach. This will be further described in considering gastric ulcer. Cancer of the stomach also may give rise to hæmatemesis.

Another frequent cause of hæmatemesis is disease of the liver. To understand how this causes bleeding from the stomach, the circulation of the blood in the walls of the latter must be explained. Blood is conveyed to the stomach by means of arteries, which come from the large artery of the body, the aorta, into which it is sent from the heart. There is this peculiarity about the veins which take the blood back from the stomach, they do not go straight into the big vein, the inferior vena cava, which empties itself into the heart, but they go into a vein called the portal vein. The portal vein, having collected blood from the stomach and intestines, is distributed to the liver, from which it is returned into the inferior vena cava and so to the heart. The substances digested in the stomach and intestines are thus carried directly to the liver. Some substances, passing readily from the intestines into the blood, act injuriously on the liver. This is the case with alcohol, especially if it is taken on an empty stomach and in excess. Alcohol brought to the liver produces a change in its structure, and its active part becomes diminished, and the firmer inert material, the kind of packing which surrounds the active part, becomes increased, and in consequence, presses upon the blood vessels, preventing the blood from flowing as easily through them. That is to say, there is a block in the liver, and the blood cannot return freely from the stomach, although it is receiving the full amount; in consequence, there must be an excess of blood in the walls of the stomach; that is to say, there is congestion. The vessels in order to get rid of the excess of blood bleed into the cavity of the stomach. Thus hæmatemesis arises from those diseases of the liver in which there is some obstruction to the flow of blood in the portal vein. There are other causes besides alcohol for obstruction in the portal vein; for example, cancer may be formed near it as it is passing into the liver, and press upon it. Or it may be that in disease of the heart there is obstruction to the emptying of the inferior vena cava, and so there will be too much blood in the liver, and the portal vein will not be able to send on its blood, and the result will be the same as when the obstruction primarily occurs in the liver.

Further, hæmatemesis occurs in cases in which there is some change in the blood, causing it to ooze through the walls of the veins or capillaries, as is the case in scurvy. Again, the walls of the arteries may be diseased. In old people the arteries that can be felt, as the radial at the wrist are often recognised as being hard, so that they can be rolled under the finger. A similar change may occur in the arteries of the stomach. In this condition arteries are very apt to rupture; then in the case of the stomach arteries there would be an escape of blood into the stomach, followed by vomiting.

It is important to come to a correct decision as to the cause of the hæmatemesis, as cases may occur in which a nurse may have to take measures to relieve it until medical aid can be obtained. When there is hæmatemesis, whatever is the cause, the patient must be kept absolutely quiet in the recumbent position, with the head rather low. No food should be given by the mouth, but the patient may suck small pieces of ice. If there is faintness it is better not to give brandy, which is likely to increase the vomiting and the amount of bleeding by its stimulating action on the heart. "Salts" may be held to the nostrils, and, if necessary, brandy can be given in an enema. The ice-bag, or, if this be not handy, a sponge-bag, partly filled with ice applied to the epigastrium in some cases is of use. With regard to drugs, gallic acid, grains x., with ten or fifteen minims of dilute sulphuric acid, will probably be ordered every two or three hours or oftener; or acetate of lead, two grains in a pill. Oil of turpentine, twenty or thirty minims, has been used, but requires care in administration. The after treatment must rest entirely in medical hands. If the hæmatemesis is due to portal congestion, then that must be lessened, and the way this is commonly done is by ensuring a free action of the bowels. The treatment of gastric ulcer will be considered later.

### Wants and Workers.

Can anyone recommend a free home for a week-minded, but harmless, woman of 33? Particulars can be obtained from M. L. A. W.

### Women's Work.

### FEMALE MEDICAL AID FOR THE WOMEN OF INDIA.

By Mrs. Scharlieb, M.D.

(*Read at the Women's Conference at Leeds.*)

### III.—THE TRAINING NECESSARY AND PROCURABLE.

The medical education of women has developed rapidly in the last few years, and whereas in 1878 there was but one medical school (the London School of Medicine for Women) and two examining bodies, there are now in the British Isles eight medical schools in which women can obtain a qualifying education, and there are six examining bodies which grant them degrees or licences. The training offered is complete, but it is long and severe, taxing to the utmost both mental and physical fitness for work. It cannot be too generally understood that the curriculum for women and the examination for qualification are identical with those of medical men, so that the women we send out are fully equipped for their work. In many instances they choose to spend additional time in post-graduate work here or at Vienna.

### The Agencies Whereby the Workers Can be Put in Relation With the Work.

Of these the largest and best developed is the grand scheme which originated with the Queen herself, and was made practical by the earnestness and business capacity of Lady Dufferin. This organisation is briefly known as "The Countess of Dufferin's Fund" or "The National Association for Supplying Female Medical Aid to the Women of India." It was started by Lady Dufferin as soon as she arrived in India, and has developed rapidly.

The objects of the Association are: (1) To bring the aims of the National Association before the British public; (2) to raise funds; (3) to select such lady doctors as may be required for the Association in India. The fund employs three grades of medical women, who in a way may be compared with the officers, non-commissioned officers, and privates of an army. The officers are termed lady doctors of the first grade. These are all fully qualified, and up to the present time have been selected from women holding degrees or licences to practice registerable in England. The second grade are styled assistant surgeons, and are composed of women educated in the medical schools of India. The third grade are the hospital assistants, principally native women. This third grade is a very important one, and, to quote from the speech of the Hon. C. H. Moore, at the annual meeting in Calcutta, it is the nucleus of a "Dufferin corps of small practitioners, equal in numbers and capacity to cope with house to house work on a scale which, if my imagination does not mislead me, would be able to reach the very core of our subject, and in comparison with which the work done to date, good and great as it is, would seem but the shell." This is doubtless true. To carry home the necessary aid to every native woman and child we need an army of native women practitioners of every caste, speaking the languages and understanding the customs of their patients. Being born in the country, and less affected by the heat and necessary discomforts of the work, they could take sole charge of cases of slight illness, and of normal labour; while they could call to their assistance in case of need the more fully qualified women of the first or second grade under whose directions they would work. When we consider the enormous mass of the female population of India—when we remember how scattered and difficult of access are the villages, it is evident that the entire produce of all the British medical schools for women would be unequal for many years to come to supply their needs. Also when we remember the poverty of the masses of the people and the small value they set on life, especially on the lives of women and children, it is evident that we must supply medical aid gratis, and look for no pecuniary return direct or indirect. On this point the Hon. Mr. Gurndas Banerjee says, "It is only by preparing a large number of female practitioners born in this country (India), versed in its customs and belonging to its people, that the Association can ever hope to supply to the mass of Indian people that medical relief which it is its object to provide for them. . . A poor purdah woman who has not means enough to pay for her medical attendant at her house and yet has sentimental objections strong enough to prevent her from seeking relief in a hospital, is more helpless and therefore more deserving of charitable relief than one who is not so hampered." No doubt there are many such women both Hindu and Mahommedan, and it will take years, nay generations, of education to enable them to overcome their prejudices and come willingly to hospital. Secondly, if they were willing to come, how would it be possible to provide hospital accommodation for such numbers? On the other hand this sort of work is very trying and unsuitable to European women.

(*To be continued.*)

### Nursing in the United States.

### TRAINING SCHOOL FOR NURSES OF THE NEW YORK HOSPITAL.

### The Nurses' Home.

In connection with one of the oldest and richest hospitals in the city of New York—the New York Hospital—is a training school for nurses that, in its way, is a model of what a training school should and may be.

The Nurses' Home is separated entirely from the hospital, although in the same grounds; so near, that a few steps will carry one from the Hospital to the Home, and yet the pupil-nurses are, for the time, isolated from all sounds and smells of hospital life, and while in their Home, have many privileges and comforts. The latter is a red brick structure, eight stories in height, and fireproof. An elevator runs from nine a.m. to ten p.m., so that the nurses do not have to use the staircase during those hours. Upon the second floor is a suite of rooms belonging to the directress of the nurses, and also the library and parlour for the nurses, where they read, use the piano, or entertain friends when off duty.

The nurses' apartments are in suites of three rooms. Every nurse has a bed-room to herself, and a reception-room between every two bed-rooms. The rooms are all well-furnished. The bed-room contains an iron bedstead, bureau, and wardrobe. The reception-room is furnished with a lounge, chairs, a writing-desk, table, and book-case. Rugs lie upon the floors, and the nurses are permitted to arrange whatever pictures and bric-à-brac they choose to bring. The building is heated by steam throughout, and lighted by electricity. The bath-rooms have porcelain tubs, and all other modern improvements.

Over the entire building there is a roof, with a canopy, and during the summer months rows of hammocks are suspended there. From this height the nurses have a fine view, and breathe fresh, pure air.

The day nurses go on duty every morning at a quarter to seven, and remain on until seven p.m. They have during this time one hour of recreation, arranged for by the head nurse (or nurse-in-charge) of each ward, and half an hour is allowed for each of the three meals.

During the two years' training four months are passed by the pupil-nurse on night duty. The hours then are from a quarter to seven p.m. to seven a.m. Three months are also passed in a thorough course of training at the Sloane Maternity Hospital, the privileges of which are extended to but two hospitals, the New York Hospital and St. Luke's Hospital, both of New York City.

The duties of the pupil-nurses are those to be expected in any large institution of this kind. The work is thorough, and the nurses do many of the dressings in the surgical wards.

The pupil-nurses are all under the supervision of a directress, chosen because of her wisdom, capability, and experience.

There are classes in materia medica, nursing, and physiology once a week, and lectures during the course by distinguished physicians and surgeons.

Oral examinations are held every six months; the questions are asked by the directress of the nurses and by the governors of the hospital board, while the final examination is conducted by one of the attending surgeons, and by one of the attending physicians.

Every Thursday evening the nurses assemble in the parlour, where a service is held, conducted each week by a different clergyman of the city. It is arranged that every Sabbath each nurse can have half a-day off duty, and there is also a half-day for each nurse during the week.

A vacation of two weeks each year is allowed to every nurse, and this is generally spent in her own home.

The costume of the New York Hospital nurses is a gingham gown with wide checks of a darker blue. This is made quite plain. A large white mill handkerchief is worn about the neck, white linen cuffs at the wrist, while a white cambric apron and white mull cap complete the costume.

### The New York City Training School for Nurses, Blackwell's Island.

THE last annual report of this celebrated training school contains much interesting matter. The addresses given to the graduating class of 1893, are reproduced in full, and Mrs. F. Rhinelander Jones' speech touches on many points of vital interest to nurses. Amongst other things, she insists most strongly that "the physician and nurses cannot be too careful to keep themselves healthy in mind and body." When a nurse is off duty it is not enough that she should have physical rest; she ought to amuse herself . . . . . Between her cases she should give her mind as well as her body good and varied food, and while at work she should try, if possible, to see a newspaper for a few minutes each day, to keep in touch with the healthy outer world."

An unused pavilion has been connected with the Nurses' Home by a covered corridor, and additional accommodation secured by its conversion into bed-rooms, class-rooms, &c. Seventy nurses have now comfortable quarters, and supply a staff to the City, Maternity, Gouverneur, and Harlem Hospitals.

A school registry on the co-operative plan is a distinguishing feature of the N.Y.C.T.S., and its first annual report is a most satisfactory one: Miss Darche herself describes it as "a society for mutual protection. It aims to exclude all graduates who prove themselves unreliable and untrustworthy, it excludes outsiders who are only partially trained, and who, in many cases, do not deserve the name 'trained nurse.' The scale of charges to patients is definitely fixed by the Registry Committee."

As regards the applicants for training as professional nurses in the N.Y.C.T.S., we find that they are admitted from 21 to 25 years of age, and have one month's probation, at the end of which the Superintendent decides as to their fitness for the work. They are on duty from 7.30 a.m. to 7.30 p.m., with an hour for dinner, and when hospital duties permit, additional time for rest and study. They are also given a half-day every week, and when possible, every second Sunday. A vacation of two weeks is allowed each year.

### Nursing in Switzerland.

By DR. CHARLES KRAFT, Director.

### PRINCIPLES OF LA SOURCE.—III.

### OUTLINE OF A DAY'S WORK.

THE various occupations of the pupils are apportioned to them in series of three weeks. One pupil appointed to the kitchen service learns there how to prepare special dishes for the sick. Another does household work. Two or three are busy in the clinic, whilst their companions visit the sick in the town, accompanied by the externes, at the wish of the latter. The clinic, I may add, rarely admits externes to its service. Two large dormitories accommodate the internes. The head nurse of the clinic occupies a special building. At a quarter-past six in the morning all are astir. The pupils make up their beds, arrange the rooms of the patients, and attend to different household details. At half-past seven breakfast; at ten minutes to eight the Director reads family prayers, at which all assemble—a chapter from the Bible, a few verses of a hymn sung, a prayer. Such is the preparation for the duties of the day. From eight to nine theoretical instruction. The Director, after having questioned the pupils upon the lessons given the day before (both theoretical and practical), and being assured that they have been understood, distributes the work for the day, designates the visits to be made in the town, and orders (and explains) the treatment for each patient. The pupils take advantage of this opportunity to ask questions or to speak of any point which puzzles or embarrasses them. This conversation lasts perhaps a quarter of an hour, and then follows the lecture. The Director then speaks slowly enough for the pupils, the most of whom have not much facility of composition to take complete notes. He illustrates his remarks on the blackboard by way of making the lectures more comprehensible.

Nine o'clock: Each pupil goes to her own particular work. Broom and brush in hand, the one assigned to the house, sweeps stairs, corridors, dusts and brushes. The kitchen assistant goes with the cook to market or prepares the mid-day meal. The nurses for the town patients go to those assigned to them. If the patients are attended by the Director, he gives the nurse her orders; if they are attended by some other medical man, the nurse is responsible to him. The head nurse of the clinic and the three or four assistants appointed for this service work with the Director and under his orders until eleven or twelve o'clock, giving baths, applications of massage or of electricity, assisting at operations, &c.

From twelve till half-past twelve, dinner.

There is no fixed routine for the afternoon, save one hour devoted by the Directress to the pupils' recitations and to familiar talk, when she endeavours to inculcate the moral and religious principles which will fortify them for the exigencies of their future vocation. This time having elapsed, the town nurses return to their patients, the others to the clinic, others write out their notes under the supervision and with the aid of the Directress.

Five o'clock: A second visit made to the sick by the Director gives him an opportunity to notice care given them by the pupils.

Half-past six: Supper.

Eight o'clock: Prayers read by the Director.

Half-past nine: Bed.

The nurses of the town take night duty in turn with the sick poor who are seriously ill or alone. The pupils of the clinic also do night duty in turn, while their companions take a rest well merited by the work of the day, always full and often very fatiguing.

(*To be continued.*)

### Nursing in Japan.

By a Travelling Nurse.

It may interest some of my fellow-workers to hear a little more about hospital life in Japan. In the Japanese Government hospitals there are now no European women employed in the wards. I heard of an English sister being in one, but she did not remain long, and at present there appears to be a growing determination on the part of the Japanese authorities to advance their own people by putting them in posts of honour and responsibility wherever it is feasible. However, much remains to be done before these hospitals can in any way compete with a model English institution in ventilation, order, and general comfort. There appears no effort to entertain the patients, who look weary and woebegone. A little diversion would break the monotony of the days spent in hospital, and possibly hasten convalescence. The absence of flowers from the wards, and especially the lack of books, pictures, or other decorations, impressed me very forcibly. The only pretty objects were the little coloured tea-pots standing by nearly every bedside. Prevalent ideas regarding digestion differed widely from those held by most English physicians, for tea seemed to be a continual feast; it was served so many times during the day to the patients, who sipped it with evident relish, judging by the noise they made in drinking it. I could not help picturing to myself the improvements which could be made by the addition of flowers and other objects of brightness and interest, so pleasant to the eyes of sick people. Japanese women are the attendants in the female wards, apparently all of the rougher classes; evidently the ladies of Japan have not yet interested themselves in the art of nursing. There was a great lack of that indescribable atmosphere of home-like comfort that reigns in hospital wards at home, where education and refinement rule supreme. I must say the hospitals appeared beautifully clean, and a negative sort of prevailing kindliness suggested itself.

Many of the nurses trained in these hospitals go out to nurse paying patients amongst the Europeans, and on every side they are well spoken of—perhaps partly because there are few other nurses, but at any rate the doctors were unanimous in expressions of approval concerning the obedience and trustworthiness of many of the natives when in charge of private patients. They are contented to do much menial work, and receive remuneration that English nurses would look upon as beneath contempt.

In the Japanese Government Hospital the surgical treatment seemed to be much the same as in ours, and the results were decidedly satisfactory.

Of course I speak only of Japanese Government hospitals, and not of such institutions as the Naval Hospital in Yokohama, and others, but in those also there are no European women nurses.

In Tokio there are lady nurses in the mission under the Bishop of Japan.

In Kioto there is a beautiful hospital belonging to the American mission, where our energetic American sisters are well to the fore, being under the direction of a fully-trained American lady, to whose courtesy I am indebted for a very happy and interesting visit.

There was a general air of comfort and contentment, the invalids looked cheerful, and in going round with the Lady Superintendent it was easy to see the good tone of the place. The nurses were evidently being trained without being antagonised, and a good feeling existed between patients, nurses, and the authorities, whilst as much liberty was allowed as order and decorum permitted.

The staff of Japanese nurses received lectures and instruction from the Lady Superintendent, and they appeared healthy and intelligent, many of them having the peaceful, contented look of those who worked from high motives, not only for a living. Perhaps many of them come straight from the mission training school, and therefore have been under good influences. These women showed what can be made out of Japanese workers when rightly handled.

The wards were strictly clean, pretty, and in beautiful order, and the mission seemed likely to become an influential centre.

Those beautiful lines of Whittier's came into my mind, for this hospital showed how well the spirit of them could be carried out:—

The paths of pain are thine, go forth
With patience,trust, and hope;
The sufferings of a sin-sick world
Shall give thee ample scope.
Besides the unveiled mysteries
Of life and death, go stand
With guarded lip, and reverent eyes
And pure of heart and hand.

### Cottage Hospitals in the West Country.

### LYME REGIS.

By Viator.

Having recently spent a few days in the ancient and historical town of Lyme Regis, a quiet place with five or six miles of hills "which can't be turned, and wouldn't pay for tunneling," between it and the nearest railway station, I took an opportunity of visiting the cottage hospital, which has been for many years a blessing to the sick and disabled of the town. It is a building of the villa type, a little way out of the town on the Seaton road, with nothing—save the inscription on the front—to distinguish it from a private house. The Nurse-Matron (who looked the personification of quiet capability) readily consented to let me see the wards, consisting of two four-bedded rooms for male and female patients, and a small room on the ground floor, originally intended for out-patients' dressings, but now provided with a bed and used for isolation cases. There were only two patients, both of whom had been inmates for many months. Their evident contentment, and the progress they had made from conditions which must have seemed almost hopeless when admitted, were testimonies to the usefulness of the institution and the good treatment they had received. The large bay window of the female ward had a nice green field beneath it, and beyond that a sea-view extending as far as Portland. A box ottoman fitted to the window served the double purpose of a very comfortable seat, and a locker for clothing. I learnt that, when hospital work permitted, the Matron did district nursing in the town, besides providing dinners for the sick poor, for confinement cases, &c. Truly a variety of work to be got out of a "one horse shay." However, she seemed to enjoy it, and only lamented the loss of a musical-box, which had given great pleasure to her district cases, but had never returned after being sent away for repairs. If any one who reads this has a musical-box to spare, I do not think he or she could do better than send it at once to Miss Eger. A pretty organette is a great source of pleasure to the in-patients. I took leave with regret, and when I departed from Lyme, on the top of a three-horse coach (the animals galloping up hill and down with equal indifference), it was with the conviction that if struck down by sickness or accident, there was no place I should prefer as a haven of refuge to the cheerful and pleasant Lyme Regis Cottage Hospital.

### Everybody's Opinion.

[Correspondence on all subjects is invited, but we cannot in any way be responsible for the opinions expressed by our correspondents. No communications can be entertained if the name and address of the correspondent is not given, or unless one side of the paper only be written on.]

### UNIQUE TESTIMONIALS.

"A LATE MATRON" writes: In reference to a paragraph in "News from the Nursing World," headed "Unique Testimonials," would it not be advisable that the Guardians of every union should be compelled by the Local Government Board, before appointing any nurse, to apply to the matron and medical officer of the hospital or infirmary where the nurse was last employed? I have heard of several cases where candidates have produced testimonials, [supposed to have been given by matrons, which proved to be merely written by friends of the nurses. In other cases nurses have obtained appointments because they possessed testimonials from one of the honorary physicians of their last hospital, he having been attended by the nurse on his regular visits round the ward. These visits possibly occupy about half an hour, therefore a testimonial given from such intercourse, without consultation with the medical officer or the matron, could have but little real value. I also have sometimes received testimonials sent in by a nurse with her applications, but I always made a point of communicating direct with those who had given them.

### ASYLUM v. HOSPITAL TRAINING.

Miss H. MORTEN writes: Dr. Harding seems to have gathered from your paragraph of November 18th that the Nurses' Co-operation has finally decided not to accept mental nurses of asylum training only. This is not so. So far, the matter has only been before a sub-committee, which has to report to the General Committee in January, the whole subject coming finally before the annual meeting in February. I have a list of fourteen public associations which have undertaken to recognize and support the three years' certificate about to be given by the Northampton County Asylum. When the list is further augmented I will send it you for publication.

### LEGALLY ENTITLED TO HER CERTIFICATE.

G. F. SHEPPARD writes from 10, Chester Place: In "Fairplay's" letter, printed at p. lxxvi. of the "Nursing Supplement" of your paper of 25th inst., the words occur: "Legally the nurse was entitled to her certificate in consideration of her three years' service." May I inquire whether such "legal" claim is based on any statute law, or simply on some agreement between the nurse and the Committee of the Royal Infirmary?

### Where to Go.

GREAT ASSEMBLY HALL, MILE END.—Lecture with lime-light dissolving views on "The Temperance Pocket Book" by Mr. Campbell, N.T.L., at eight p.m., on December 4th. Admission free. Music from half-past seven to eight.

GREAT ASSEMBLY HALL, MILE END.—Lecture on "Brain and Nerve" by Dr. Andrew Wilson, on December 7th, at eight. Organ recital by Mr. Day Winter before the lecture.

### Death in Our Ranks.

NURSE ALICE WATSON died at Rotherham Union Infirmary on November 14th, of enteric fever. She had held the post of Head Nurse for two and a-half years, and was universally respected in the infirmary, where her death is deeply regretted.

### Medico-Psychological Association of Great Britain and Ireland.

### WRITTEN EXAMINATION FOR NURSING CERTIFICATE, NOVEMBER, 1893.

1. What is the ordinary temperature of the body in health? Is elevation of temperature frequent amongst the insane? How would you proceed to ascertain exactly whether there was any rise of temperature in a given case?

2. A patient has had a fall; what symptoms or conditions would lead you to suppose that one or more ribs had been broken?

3. An epileptic patient has received a deeply-incised wound of the scalp, which is bleeding freely; describe the steps you would take to arrest the bleeding and temporarily dress the wound.

4. What steps would you take to prevent the occurrence of bed-sores in an enfeebled patient confined to bed? State the parts of the body to which you would pay special attention.

5. What indications in a patient's condition would lead you to suppose that insufficient nutriment was being taken, and that special attention to alimentation was necessary?

6. Describe generally the mental and bodily states of a patient suffering from acute melancholia, and how would you act towards such a case if you were placed in special charge?

7. Give the anatomical names of the following bones: (*a*) Collar bone, (*b*) shoulder blade, (*c*) thigh bone, (*d*) bones of arm, (*e*) bones of leg.

8. What weight and form of bed-clothing would you consider sufficient for patients in cold weather?

9. Amongst what class of insane patients is the suicidal impulse most likely to occur?

10. The following articles of dietary are required for a patient: (*a*) Beef tea, (*b*) arrowroot, (*c*) egg-flip, (*d*) custard pudding. How would you prepare them?

11. A patient has swallowed poison; what means are you acquainted with for emptying the stomach? Give names and doses of some simple emetics.

12. What class of patients require special attention at meals, and what precautions should be observed as to the character of their food?

Three hours allowed to answer this paper.

The questions are valued at ten marks each; two-thirds of the possible total of marks are required to pass.

ORAL EXAMINATION (ten marks for each question).

1. How would you deal with a person whom you found suspended by the neck?
2. How do you perform artificial respiration?
3. What conditions of urine are to be noticed and reported?
4. How would you deal with a maniacal patient who was inclined to be violent?
5. How would you treat a person who had fainted?
6. What would be the symptoms of such a person?
7. What precautions would you take to prevent fire?
8. How would you deal with a suicidal patient?
9. What is homicide? How would you deal with a patient so inclined?
10. What changes in the blood take place in the circulation?
11. How would you make a linseed-meal poultice?
12. What would you do with a patient who was choking?

DERBY COUNTY ASYLUM.

At the November examinations of the Medico-Psychological Association of Great Britain and Ireland, held at Mickleover Asylum, on November 6th and 7th, twelve candidates, including the two chief attendants and the housekeeper, who acts as deputy chief nurse, presented themselves for examination. The examiner was Dr. E. Whitcombe, Superintendent of Birmingham Asylum, an ex-President of the Association. The twelve successful candidates are Harry Bird, George Henry Twigg, Henry Hutchinson, Thomas Travis, Jesse Speck, James Plummer, Thomas Cooper, Annie Newport, Mary Withers, Ada Elizabeth Savidge, Mary Ellen Fletcher, and Elizabeth Hall.

### In the Canaries.

(By a Patient.)

### SANTA CRUZ.

Having been asked for a few lines about Teneriffe, I write them with the greatest alacrity, for I owe a debt of gratitude to one of the hospitals there.

It is seven years since I was unexpectedly landed at Santa Cruz, Teneriffe. I was on my way to the Cape, when the ship's doctor decreed that I was to be dropped, and in answer to my protestations, brusquely said that the hospital in Santa Cruz was the only chance I had of being saved from becoming food for the sharks in the Atlantic.

I had had a severe attack of influenza followed by pneumonia, in September, 1886, and my lungs remained in a delicate condition, so I was ordered to Natal for the winter months. Feeling wonderfully well the first day on board, I forgot or ignored my doctor's strict instructions as to wrapping up well, &c. My foolhardiness was punished by another attack of pneumonia. So after kind good-byes and words of encouragement from my sympathetic fellow-travellers I was lowered into the health-officer's boat, and the heaving of the anchor of the "Spartan" somehow seemed to sever the last link that bound me to home. As in a dream I heard soft voices saying "Pobrecita" (poor child), then a chorus of strange sounds greeted my landing at the pier, and I remembered nothing more.

The sunshine was pouring into my room, and lighting up all its corners, when I awoke from what seemed like prolonged sleep to find myself in the hospital of Santa Cruz. I had scarcely time to look about me and take in my surroundings, when a Sister of Charity in a wonderful head-dress came up to my bed and, making the sign of the cross on my forehead, spoke quickly in Spanish. From her glad expression I guessed that she thought me better. Then came in the kind old doctor, Senor Don Diego Costa, a specialist in lung complaints, whose promise in French of a speedy recovery with care and good nursing fell gratefully on my ears.

The happy weeks of convalescence that followed can be of little interest to my readers, though full of pleasing reminiscences to myself. The daily French chat with the doctor, the little bunch of fresh violets every morning brought by a sweet little mite who had been found by the sisters deserted and half dead in a cave, and, lastly, the visits of the dearest old Spanish lady in the world, Donna Rafaela Cambreleng, whose name I can never mention without gratitude, was my staunchest friend, and her death, in May, 1892, was as great a trouble as the loss of my own parents. Donna Rafaela was an active old maid of 72, full of energy and fun, wrapped up heart and soul in the hospital, into which she constantly trotted to see if she could be of help. She would as willingly attack an American millionaire for a donation, as buy a new ribbon for the foundlings' hats.

Many were the bazaars and raffles she got up to eke out the narrow funds of the hospital. The provincial corporation are supposed to pay the sisters £2,000 a year, but plenty of excuses are always forthcoming for not paying more than the half, and a change of Spanish ministers usually means a half year's delay in paying the poor sisters. If it were not for generous patrons, among whom several English names figure honourably, it would be impossible to keep up the establishment.

It is more like two or three hospitals joined together than a single edifice. One wing is entirely devoted to patients, there being accommodation for about 120 men and 100 women. Another is given up to the foundlings, whose long, exquisitely clean rooms are filled with rows of cots. These little unfortunates are tenderly looked after, and find mothers in the good sisters. Many are boarded out in peasants families, who are responsible for their health and well-being, and are obliged to bring their charges once a month to the hospital to be inspected by the sisters. In many cases the peasants adopt them, otherwise the child returns to the hospital when seven years old, and then receives a fair education. The boys later on are taught a handicraft, and the girls go out as servants, or remain at the hospital if they prefer it. In the latter case, the girls learn fine embroidery, lace work, and every kind of fancy work. I brought back some handkerchiefs beautifully embroidered by them which cost only one shilling each. The sisters are only too pleased to receive orders for these, or embroidered pillow cases, sheets, &c.

Another wing is devoted to the imbeciles, who are looked after by a young sister. No lepers are received in these establishments, there being a general hospital for them in Las Palmas, where they receive special treatment by tepid baths, &c. In 1884 part of the hospital was burnt down through the carelessness of a servant, and not being insured, the reconstruction was a costly matter for the sisters. In two months' time I was strong and well, and quite able to continue my voyage to Natal, but Dr. Costa's advice was "locus sta," and I followed it, the result being that I became a living advertisement for the climate of Teneriffe. During my illness I had been located in a nice airy little room looking to the sea, had received constant medical attendance, medicines, good beef tea, later on supplemented by wholesome food, for 3s. 6d. a day. This is the usual charge, but the sisters will make allowance for straitened circumstances and accept even less.

### Appointments.

[It is requested that successful candidates will send a copy of their applications and testimonials, with date of election, to The Editor, The Lodge, Porchester Square, W.]

The Sisters' Hospital, St. Albans.—Miss Alice Rees has been made Matron of this hospital. She was Charge Nurse at the South-Western Fever Hospital, Stockwell, and afterwards Matron of the St. Marylebone Isolation Hospital. Miss Rees has our good wishes in her future work.

Emergency Hospital, Washington. D.C.—Miss Rebecca West has been appointed Superintendent of this hospital. She has for a considerable time successfully fulfilled the duties of Assistant Superintendent of Nurses at the Philadelphia Hospital, where her departure is greatly regretted, and her place will be difficult to fill. We cordially congratulate Miss West on her promotion, and wish her a most prosperous career.

Dunster and Minehead Village Hospital.—Miss R. H. de Teissier Crosse has been made Matron of this hospital. She was trained at the Radcliffe Infirmary, Oxford, and was for two years Charge Nurse of the women and children's ward at the Buckinghamshire General Infirmary, Aylesbury. Miss Crosse was on two occasions *locum tenens* for the matron at Abingdon Cottage Hospital, and has had experience in private nursing. We hope that every success will attend her in her new work.

Hospital for Consumption, Brompton.—Miss Charlotte Davidson has been appointed Lady Superintendent at this hospital. Miss Davidson was trained at St. Thomas's Hospital, where she afterwards held the post of Assistant Night Superintendent. She was then made Night Superintendent at Marylebone Infirmary, and was subsequently Assistant Lady Superintendent and Matron at the Royal Infirmary, Liverpool. Miss Davidson's training and varied experience have been valuable preparations for the important appointment which she has obtained, and we cordially wish her every success in her new work, and congratulate the committee at Brompton on having secured her services.

### The Book World for Women and Nurses.

[We invite Correspondence, Criticism, Enquiries, and Notes on Books likely to interest Women and Nurses. Address, Editor, The Hospital (Nurses' Book World), 428, Strand, W.C.]

The Pall Mall Magazine Christmas Number.

Amongst the mass of literature provided for our Christmas entertainment, it will be difficult to find a better outlay for the modest shilling than the "Pall Mall Magazine." Prefaced by a charming and delicately tinted portrait of a quaint little maid from far Japan, the letterpress commences with a tribute to Lord Roberts in verse from Rudyard Kipling's pen. The most of us know Tommy Atkins as he is by now, through the genius of the same writer; and so praise of Bobs, the soldier's hero, comes to us suitably expressed in his vernacular. The excellence of the illustrations which add to the attractiou of this song of praise, is carried out through the whole number. There is only one disappointment throughout the many pages, and that is that "Lord Ormont and His Aminta" is only the commencement of a serial instead of representing the complete story we look for in our Christmas numbers. It is bad enough to have to suspend our interest for a month at ordinary seasons of the year, but at Christmas we have learnt by custom to look for our literary pleasures complete. Fortunately the remaining pages of the Christmas "Pall Mall Magazine" provide us with reason enough for forgetting the incompleteness of the commencement. Christmas in New Zealand takes us a mental journey to summer time. Words and pictures alike cause us to turn our backs to the steady gloom of the London day, the grinding thunder of the wheeled traffic, and the mockery of the music of the barrel organ. Only by a mental effacement of all realities can we realise that "Christmas on an idle bay, with a hundred islets; where, beneath such blue as Naples cannot boast of, exertion becomes distasteful compared with the delights of lazing away the hours in a drifting boat," can exist contemporaneously with our essentially "within four wall" season. Our imagination is helped on its flight by soft portraits of wooded heights, fair lakes and bold rockland, and reluctanty we yield to reality again, and the passing omnibus asserts itself once more. Perhaps more akin to our every day, is the glimpse at "Unknown Paris." Human nature, even in the different dress which diverse tongues lend, has so much in common, that differences only heighten the interest. This glimpse into the Paris slums gives us acquaintance with classes and scenes like and yet unlike those which in our own London, we, many of us, are familiar with Rag-pickers and beggars; amongst these our author leads us, with a skill which places very vividly the various characters and phases before us. We find ourselves stirred with a desire to establish a refuge such as "Fradin's" offers to the class of beggars who in London have no such shelter from the streets. "Fradin's," be it remembered, is no charity, and the hundreds who invade its portals at eleven each night pay the required twopence in advance for a cup of soup, some bread, or a glass of wine, and such shelter and rest as the barn-like building, with its bare floor and benches, affords. Whilst rag-picking is allied to a certain amount of self-respect and honour, and a hardly-earned bare sufficiency, the begging trade appears to be carried on under an organization of artistic fraud, which the London brethren have not yet attained to. The bureau of information which supplies particulars and characteristics of households in the giving of alms is, apart from the sad aspect, certainly an amusing study. The unsuspecting house-

**THE BOOK WORLD FOR WOMEN AND NURSES**—*continued.*

holder, if he could look within the records, might find himself described as "Old Radical, very rich. Go as victim of the priests," or in some other concisely critical manner. All the pages of this article are interesting and instructive, and though complete in itself, we are glad that we have not said good-bye to an excellent conductor, who knows how to shed light on the paths where he leads us. "The Confessions of An Interviewer" make us wish that if we must have interviews, they could all be served in the way Mr. Lane has given us this pot pourri of reminiscences. In a few telling strokes, he places his "subjects" before us more effectually than by pages of the most skilful question and answer system ever can do. Interesting as the articles we have mentioned must prove to most readers, we must not omit to assure the lover of fiction that "The Mystery of the Hacienda," by Bret Harte, is as keenly interesting as most of this author's sketches. Like the rest of the contents, it is profusely and delightfully illustrated.

Everybody's Book of Acting Charades. By J. J. Pledge, F.R.G.S. (Saxon and Co., London.)

This little volume, in appearance resembling "Everybody's Pocket Encyclopædia," will form a popular and useful Christmas present for holiday folk. Mr. Pledge has realised and supplied a want for really simple dramatic entertainments. Most small parlour plays have hitherto been published separately or in too expensive a collective form. Separately, the difficulty of choice presents itself. Here, for a very small cost, is a little book with numerous charades simply and practically arranged, which when exhausted will serve as models for original efforts on the part of the actors at the expenditure of a very small amount of ingenuity.

